# Double burden: financial toxicity in patients with advanced soft tissue sarcoma at the start of first-line palliative chemotherapy: baseline data from the HOLISTIC study

**DOI:** 10.1007/s00520-025-09248-5

**Published:** 2025-02-27

**Authors:** E. Roets, E. Younger, R. L. Jones, D.den Hollander, I. M. E. Desar, R. J. Young, A. W. Oosten, J. J. de Haan, H. Gelderblom, N. Steeghs, W. T. A. van der Graaf, O. Husson

**Affiliations:** 1 Department of Medical Oncology, The Netherlands Cancer Institute, Plesmanlaan 121, 1066 CX Amsterdam, The Netherlands; 2 Sarcoma Unit, Royal Marsden NHS Foundation Trust, Fulham Road, London, SW3 6JJ UK; 3 Division of Clinical Studies, Institute of Cancer Research, London, UK; 4 Department of Medical Oncology, Radboud University Medical Centre, Nijmegen, The Netherlands; 5 Academic Unit of Clinical Oncology, The University of Sheffield, Sheffield, UK; 6 Department of Medical Oncology, Erasmus MC Cancer Institute, Erasmus University Medical Center, Rotterdam, The Netherlands; 7 Department of Medical Oncology, University of Groningen, University Medical Center Groningen, Groningen, The Netherlands; 8 Department of Medical Oncology, Leiden University Medical Center, Leiden, The Netherlands; 9 Department of Surgical Oncology, ErasmusMC Cancer Institute, Erasmus University Medical Center, Doctor Molewaterplein 40, 3015 GD Rotterdam, The Netherlands

**Keywords:** Sarcoma, Quality of life, Financial toxicity, Cost, Employment

## Abstract

**Purpose:**

The HOLISTIC study assessed health-related quality of life (HRQoL) in advanced soft tissue sarcoma (STS) patients receiving first-line palliative chemotherapy. The secondary objective discussed here is to evaluate baseline self-reported financial difficulties and associated sociodemographic factors and global health status (GHS), compare financial toxicity between patients in the United Kingdom (UK) and the Netherlands (NL), and evaluate the consequences of financial toxicity.

**Methods:**

This prospective study included 72 UK and 65 NL patients. Financial toxicity was evaluated by the financial difficulties scale of the EORTC QLQ-C30. Associated factors (i.e., country, gender, educational level, relationship status, employment changes, income, age, time since diagnosis, and GHS) were analyzed using descriptive analysis, Chi-square tests, and univariate and multivariate logistic regression.

**Results:**

Median participant age was 62 (range: 27–79) years, and gender distribution was equal. 58% of UK and 48% of NL patients had no income or a monthly income ≤ £/€ 2000 (*p* = 0.417). Self-reported additional costs for medication (31% vs. 9%, *p* < 0.001) and parking (75% vs. 41%, *p* < 0.001) were more prevalent among Dutch than UK patients. Travel expenses were similar: 68% in NL and 66% in UK. Univariate analysis showed an increased risk of financial toxicity in UK patients (40% vs. 22% [NL], *p* = 0.023), single patients (52% vs. 27% [with partner], *p* = 0.014), and those with a change in employment status (46% vs. 24% [no change], *p* = 0.019). In UK patients, multivariate analysis indicated lower odds for financial toxicity for patients with a high income (OR 0.207, *p* = 0.031) and higher odds for patients with a worse GHS (OR 5.171, *p* = 0.012), whereas in NL, higher odds were seen for male (OR 13.286, *p* = 0.027) and single (OR 41.735, *p* = 0.007) patients.

**Conclusion:**

Financial toxicity was common among advanced STS already at the start of palliative chemotherapy, influenced by factors such as residence country, income, relationship status, gender, and GHS. Timely interventions are needed to address financial challenges in this population.

## Highlights

- STS patients at the start of palliative chemotherapy already experience financial toxicity, affecting their quality of life.
- Financial toxicity scores are higher in the UK than in NL.
- Financial toxicity is influenced by factors including residence country, income, relationship status, gender, and GHS.
- Predictors of financial toxicity differ between UK and NL patients.
- Toxicity scores should include information on the financial burden of advanced STS patients starting palliative chemotherapy.

## Introduction

Soft tissue sarcomas (STS) are a group of rare, heterogeneous tumors of mesenchymal origin. Approximately 10% of patients present with metastatic disease, and around 50% of patients with initially localized (intermediate or high-grade) tumors will eventually develop advanced disease [1]. Palliative systemic therapy is the mainstay of treatment for patients with advanced STS. Anthracyclines have been the standard first-line treatment since the 1970s [2, 3].

Cancer patients are often confronted with financial challenges as a consequence of their disease and its treatment, which is referred to as “financial toxicity” [4]. Financial toxicity in cancer patients is increasingly recognized as a factor that may negatively impact quality of life (QoL) [5–7]. Several studies have examined the prevalence of financial toxicity among long-term cancer survivors. While there is some data on financial toxicity in patients receiving palliative therapy, much of this literature originates from countries without a public healthcare system or a mixed system with public and private providers, where out-of-pocket costs are often higher [8–13]. Additionally, many studies focus on different cancer types or disease settings (e.g., localized disease, cancer survivors), and many of these studies are outdated, having been published before 2000 [14–16]. Little is known about patients’ experiences of financial toxicity in a palliative treatment setting in countries with public healthcare systems, such as the Netherlands and the UK, and financial toxicity in advanced STS patients has not been evaluated thus far. This population is particularly relevant to study because STS are rare cancers that require optimal care to be delivered in specialized reference centers. As a result, patients may need to travel long distances repeatedly for consultations, treatments, and follow-up appointments. This can lead to significant travel-related expenses, such as fuel, public transport fares, parking fees, and sometimes overnight accommodations. Moreover, due to the rarity of sarcomas, some innovative (targeted) treatments or specific supportive care measures may not yet be covered by standard insurance schemes or national health systems. These factors could contribute to financial toxicity, especially in the palliative setting, where experimental or off-label treatments might also be considered.

In the United Kingdom (UK), the healthcare system, known as the National Health Service (NHS), is primarily funded through taxation, and patients do not pay directly for most services at the point of use. Conversely, in the Dutch healthcare system, operating on a combination of private and public financing, residents pay for mandatory health insurance provided by private insurers, with optional extra packages. Therefore, in the Netherlands (NL), out-of-pocket costs vary depending on the level of coverage within the insurance plan. Moreover, in the Netherlands, patients first have to pay the “own risk” amount, which is an annual amount paid out of pocket for treatments and medicines, before health insurance will cover the rest. While in both the UK and NL, direct treatment costs (e.g., hospital stay) are absorbed by the health system, indirect treatment-related costs (e.g., travel and parking expenses, reduced employment, paying for help at home) are paid out of pocket and can possibly lead to financial toxicity [17]. Multiple studies have demonstrated an association between health-related quality of life (HRQoL) and financial toxicity in long-term cancer survivors [15, 18]. Other factors, such as employment status or income, might influence financial toxicity but are often not included in studies evaluating financial toxicity [19]. Moreover, for other cancer types, it has been demonstrated that financial toxicity might reduce therapeutic compliance and may influence treatment decisions [20–22].

The prospective HOLISTIC (health-related quality of life in patients with advanced soft tissue sarcomas treated with chemotherapy) study included patients aged ≥ 18 years receiving first-line palliative chemotherapy for advanced STS in the UK and NL (NCT03621332). The study protocol and first results on patients’ priorities and preferences were reported earlier (13,14).

Here, we present a secondary analysis of the results of the HOLISTIC study, which aims to assess the prevalence and associated factors (i.e., sociodemographic, direct and indirect costs, and employment status) of self-reported financial toxicity among patients with advanced STS at the start of palliative chemotherapy. Secondary aims encompass a comparison of the prevalence of financial toxicity between patients in the UK and NL and the evaluation of the consequences of financial toxicity.

## Material and methods

Ethical approval of the HOLISTIC study was obtained in the UK and NL. The study was carried out in accordance with the Declaration of Helsinki. Patients were identified by the responsible medical oncology consultant, who introduced the study to potential participants and provided an invitation package containing a link to a secure website, where interested patients could provide informed consent and complete the questionnaires online. Alternatively, patients were also given the option to complete the questionnaires on paper if preferred. Data were collected using the patient-reported outcomes following initial treatment and long-term evaluation of survivorship (PROFILES) registry [23]. Participants completed electronic or paper questionnaires before starting first-line chemotherapy (i.e., baseline) after each cycle of chemotherapy and three months during follow-up. Patients were recruited between 2018 and 2020 from eight sarcoma centers, two in the UK and five in the Netherlands. Full details of the protocol are published elsewhere [24, 25]. This secondary analysis focuses on financial toxicity just before the start of palliative chemotherapy.

### Financial toxicity and associated factors

Financial toxicity was evaluated by the financial difficulties scale of the EORTC QLQ-C30 (“Has your physical condition or medical treatment caused you financial difficulties?”) [26]. EORTC QLQ-C30 scales were transformed to linear scores ranging from 0 to 100, according to the scoring manual [27]. Therefore, a higher score for financial toxicity translates to greater financial toxicity.

Independent variables associated with the outcome “financial toxicity” were derived from sociodemographic characteristics (i.e., country, gender, educational level, change in employment status, patients’ monthly income, age at study entry, time since diagnosis, and relationship status). Additionally, global health status (GHS) was evaluated by questions 29 and 30 of the EORTC QLQ-C30: “How would you rate your overall health during the past week?” and “How would you rate your overall quality of life during the past week?” Questions of the EORTC item library were measured as categorical variables, and responses were dichotomized as follows: “a little,” “quite a bit,” and “very much” were combined as having experienced the event (e.g., extra costs), and responses of “not at all” were considered as not having experienced the event. Extra costs refer to costs related to the patient’s physical condition or medical treatment. Income was assessed as a categorical variable with six income groups. For practical reasons, this variable was condensed into three groups (i.e., no income or net income < £/€2000, > £/€2000, prefer not to say). The cut-off of £/€2000 was chosen based on a median weekly income of £569 (gross) in 2018 in the UK and a median yearly income of €40.600 (gross) in the NL [28, 29]. Working age was defined as age < 65 years. For the question “Has your employment status changed because of your cancer or its treatment?” responses were categorized into three groups: no, yes, and not applicable (i.e., retired). Educational level was divided into three groups: low (i.e., primary/secondary), medium (i.e., vocation/college/diploma), and high (i.e., university/postgraduate) educational level.

### Consequences of financial toxicity

Secondary outcomes, including changes in lifestyle due to cancer, the effect of cancer-related expenses on treatment decisions, debts, and hindrance to work, were evaluated using questions from the EORTC computer adaptive testing (CAT) instrument. Workability was assessed using two questions of the work ability index (WAI): “current work ability compared with the lifetime best (range 0–10)” and “estimated work impairment due to diseases (range 1–6)” [30–32]. The four-item decisional conflict scale (“SURE”) was used to measure the level of uncertainty over the decision to receive chemotherapy [33]; scores ≤ 3 indicated some degree of decisional conflict.

### Statistical analysis

Descriptive statistics were used to describe sociodemographic and clinical characteristics. For the EORTC QLQ-C30 financial difficulty, scale mean score and standard deviation were reported separately for UK and NL patients. In addition, country general population norm data for the EORTC QLQ-C30 were used to facilitate the interpretation of HRQoL outcomes, including financial toxicity and GHS. For this purpose, normative data for the EORTC QLQ-C30, collected in 2017, were used, both for the UK and NL [34]. For each participant, the difference was calculated between the mean score for financial toxicity (EORTC QLQ-C30) and the mean score for financial difficulties of the norm population of the UK (mean 15, standard deviation [SD] 29) or NL (mean 5, SD 17). According to the evidence-based guidelines for the determination of sample size and interpretation of EORTC QLQ-C30, for financial toxicity and GHS, scores ≥ 11 higher or ≥ 10 points lower than the norm data were considered as clinically meaningful worse scores, respectively [35]. Hence, the outcomes of financial toxicity and GHS were dichotomized into categories of “clinically meaningful worse score” and “no clinically meaningful worse score” compared to the general population norm data.

First, univariable models with financial toxicity (EORTC QLQ-C30) as an outcome and each independent variable were created. Thereafter, a multivariable logistic regression model was constructed using backward selection. To identify differences in associated factors of financial toxicity between patients in the UK and the NL, two separate models were created. For all analyses, we used IBM SPSS Statistics, version 29.0.

## Results

### Sociodemographic characteristics

The baseline questionnaire was completed by 137 patients in the UK (*n* = 72) and NL (*n* = 65). The sociodemographic characteristics of patients in the UK and NL are described in Appendix table 4 [24]. The median age of patients was 62 (27–79) years, and gender distribution was even (male: *n* = 68, female: *n* = 69). Most patients were married or in a relationship (*n* = 114, 83%), and the majority had a medium or high educational level (*n* = 110, 81%). Most patients had metastatic disease (*n* = 125, 91%) with leiomyosarcoma (*n* = 40, 29%), liposarcoma (*n* = 30, 22%), and undifferentiated pleomorphic sarcoma (*n* = 17, 12%) as most common STS subtypes. In 57% of cases (*n* = 78), the interval between diagnosis of advanced STS and study participation was > 6 months. Bivariate analysis showed that patients in the UK more often had a timeline of ≥ 6 months between diagnosis and study participation (65%, 47/72) compared to Dutch patients (48%, 31/65, *p* = 0.041). The majority of Dutch patients had a medium educational level (75%, 49/65), whereas in the UK, most patients either had a low (25%, 18/72) or a high (33%, 24/72) educational level (*p* = 0.0001). No significant differences were seen between patients in the UK and NL for age, gender, employment status, relationship status, ethnicity, and clinical characteristics.

### Income and employment of patients in the UK and NL

At the time of study enrolment, 40% of patients (NL: 39% 25/65; UK: 40%, 29/72; *p* = 0.828) were retired. Fifteen percent of patients (NL: 19%, 12/65; UK: 13% 9/72; *p* = 0.333) were on sick leave (temporary or long term), and 6% of patients (NL: 8%, 5/65; UK: 4%, 3/72; *p* = 0.380) reported that they were disabled. Of patients of working age, 50% (19/38) and 57% (24/42) of patients in NL and UK, respectively, were full-time or part-time employed (*p* = 0.522).

**Fig. 1:**
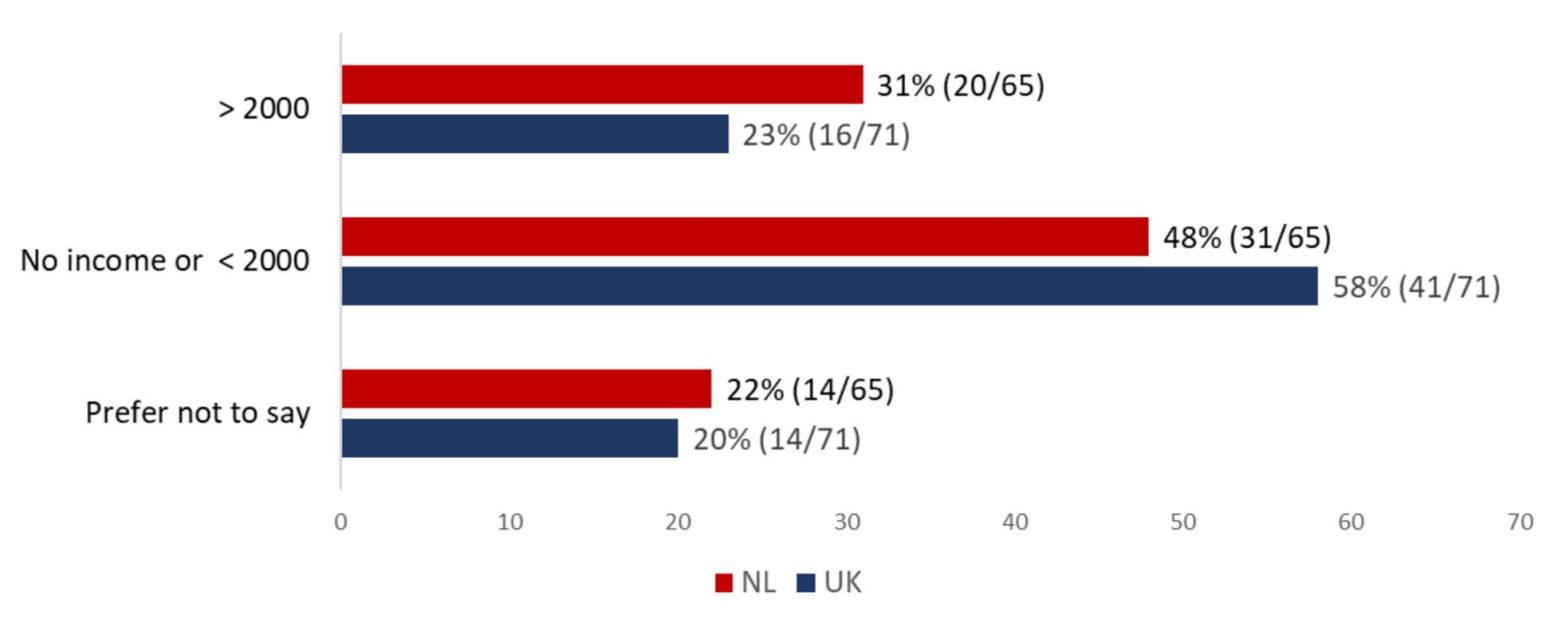
Patients net income per month in € (NL) or £ (UK). *P*-value Chi-Square test 0.456

In the entire group, 40% of patients (NL: 48%, 31/65; UK: 33%, 24/72; *p* = 0.149) reported a change in employment status due to their sarcoma diagnosis or treatment. Fifty-eight percent of patients in the UK (41/71) and 48% of Dutch patients (31/65) had either no income or a net income per month < £/€ 2000, excluding any sickness benefits (Fig. 1).

### Self-reported extra costs of patients in the UK and NL

Self-reported additional costs for medication (31% vs. 9%, *p* < 0.001) and parking (75% vs. 41%, *p* < 0.001) were more prevalent among Dutch than UK patients (Table 1). Travel expenses were frequently reported, both in NL (68%) and in the UK (66%). Overall, additional costs for medication, parking, and travel expenses led to financial difficulties in 12%, 12%, and 10% of patients, respectively.

**Table 1:** Patients reporting sarcoma-related extra costs^a^

	Total (*n* = 137)	UK (*n* = 72)	NL (*n* = 65)	*P*-value
Medication^b^	26 (19%)	6 (9%)	20 (31%)	** < 0.001**
Leading to financial difficulties	3 (12%)	1 (17%)	2 (10%)	
Travel expenses^b^	90 (67%)	47 (66%)	43 (68%)	0.800
Leading to financial difficulties^c^	9 (10%)	8 (17%)	1 (2%)	
Parking^b^	76 (57%)	29 (41%)	47 (75%)	** < 0.001**
Leading to financial difficulties^d^	9 (12%)	7 (24%)	2 (4%)	
Medical devices^e^	17 (13%)	9 (13%)	8 (13%)	0.994
Leading to financial difficulties	2 (12%)	2 (22%)	0 (0%)	
Other	7 (5%)	6 (8%)	1 (2%)	0.071
Leading to financial difficulties	5 (71.4%)	5 (83%)	0 (0%)	

^a^Responses were dichotomized as follows: “a little,” “quite a bit,” and “very much” were grouped together to indicate having experienced extra costs, while responses of “not at all” were categorized as not having experienced the event

^b^Three missing values

^c^Missing in three patients that reported extra costs

^d^Missing in two patients that reported extra costs

^e^Five missing values

### Prevalence of financial toxicity according to the EORTC QLQ-C30 and associated factors

The mean score for the EORTC QLQ-C30 financial difficulty scale was 24 (SD 34) for UK patients and 12 (SD 25) for NL patients. The results of the univariate analysis are reported in Table 2. Forty percent (29/72) of patients in the UK compared to 22% (14/65) of Dutch patients reported clinically meaningful worse scores for financial toxicity compared to the norm population (*p* = 0.020). According to univariate analysis, a higher prevalence of financial toxicity was observed in single patients (52% vs. 27% [with partner], *p* = 0.014) and patients with a change in employment status (46% vs. 24% [no change], *p* = 0.019. In multivariate analysis, in the subgroup of UK patients, factors negatively associated with experiencing financial toxicity were a net monthly income ≤ £/2000) (*p* = 0.018) and patients reporting a worse GHS compared to norm data (*p* = 0.012) (Table 3). Extra costs were not a significant predictor for financial toxicity (*p* = 0.088). In the subgroup of NL patients, negative predictors for experiencing financial toxicity were male sex (*p* = 0.027) and being single (*p* = 0.007). Change in employment status was not a significant predictor (*p* = 0.061).

**Table 2:** Univariate analysis for financial toxicity

Participant characteristics	Financial toxicity^a^				
	No	Yes	Total	Odds ratio	*P*-value
Country					**0.023**
UK	43 (60)	29 (40)	72 (100)	2.409	
NL (reference)	51 (79)	14 (22)	65 (100)	Ref	
Age, years					0.261
18–39	7 (64)	4 (36)	11 (100)	1.846	
40–65	44 (63)	26 (37)	70 (100)	1.909	
> 65 (reference)	43 (77)	13 (23)	56 (100)		
Disease timeline					0.898
≤ 6 months (reference)	40 (68)	19 (32)	59 (100)	Ref	
> 6 months	54 (69)	24 (31)	78 (100)	0.953	
Gender					0.357
Male	44 (65)	24 (35)	68 (100)	1.407	
Female (reference)	50 (73)	19 (28)	69 (100)	Ref	
Relationship status					**0.014**
Married/partner (reference)	83 (73)	31 (27)	114 (100)	Ref	
Single	11 (48)	12 (52)	23 (100)	3.213	
Education level					0.124
Low	14 (52)	13 (48)	27 (100)	2.670	
Medium	57 (72)	22 (28)	79 (100)	1.129	
High (reference)	23 (74)	8 (26)	31 (100)	Ref	
Employment status changed due to sarcoma					**0.019**
No (reference)	22 (76)	7 (24)	29 (100)	Ref	
Yes	30 (55)	25 (46)	55 (100)	2.619	
Not applicable (retired)	42 (79)	11 (21)	53 (100)	0.843	
Patients’ net monthly income					0.093
No income or ≤ £/€ 2000 (reference)	44 (61)	28 (39)	72 (100)	Ref	
> £/€ 2000	28 (78)	8 (22)	36 (100)	0.439	
Prefer not to say	22 (79)	6 (21)	28 (100)	0.419	
Any type of extra sarcoma-related costs^b^					
No (reference)	22 (76)	7 (24)	29 (100)	Ref	0.317
Yes	70 (66)	36 (34)	106 (100)	1.616	
Worse GHS compared to norm data^c^					0.086
No (reference)	62 (74)	22 (26)	84 (100)	Ref	
Yes	32 (60)	21 (40)	53 (100)	1.909	

*NL*, the Netherlands; *UK*, United Kingdom; *GHS*, Global Health Status; *Ref*, reference

**Table 3:** Multivariate logistic regression analysis of factors associated with financial toxicity^a,b^

Country	Variables	Odds ratio	*P*-value
UK	**Patients’ net monthly income**		**0.018**
	No income or ≤ £/2000 (reference)		
	> £/2000	0.207	
	Prefer not to say	0.131	
	**Worse GHS compared to norm data** (reference = no)^c^		
	Yes	5.171	**0.012**
	**Any type of extra sarcoma-related costs**^d^ (reference = no)		
	Yes	3.472	0.088
NL	**Gender** (reference = female)		
	Male	13.286	**0.027**
	**Relationship status** (reference = married/partner)		
	Single	41.735	**0.007**
	**Employment status changed due to sarcoma**		0.061
	No (reference)		
	Yes	11.748	
	Not applicable (retired)	1.866	

*NL*, the Netherlands; *UK*, United Kingdom; *GHS*, Global Health Status

^a^For each participant, the difference was calculated between the mean score for financial toxicity (EORTC QLQ-C30) and the mean score for financial difficulties of the norm population of the UK (mean 14.5, SD 28.7) or the Netherlands (mean 4.9, SD 17.1) [34]. A score of ≥ 11 points higher compared to the norm data was considered as a clinically meaningful worse score [35]

^b^The model was generated via backward selection

^c^For each participant, the difference was calculated between the mean score for GHS (EORTC QLQ-C30) and the mean score for GHS of the norm population of the UK (mean 62.3, SD 23.7) or the Netherlands (mean 77.4, SD 19.8) [34]. A score of ≥ 10 points lower compared to the norm data was considered as a clinically meaningful worse score [35]

^d^Any type of extra costs, including “medication,” “travel expenses,” “parking costs,” “medical devices,” and “other”

### Consequences of financial toxicity

Extra costs influenced treatment decisions in 8% (NL, 5/64) and 10% (UK, 7/72) of patients (*p* = 0.695). Financial difficulties led to changes in lifestyle in 22% (NL, 14/64) and 35% (UK, 25/72) of patients (*p* = 0.098). Six percent (NL, 4/64) and 17% (UK, 12/72) of patients experienced debts due to their disease (*p* = 0.060). Among the 55 patients who indicated a change in their employment status, 13% (7/55) stated that they reduced their working hours, 71% (39/55) reported being on sick leave, and 16% (9/55) reported to have quit work completely because of their sarcoma diagnosis or treatment. Among 45 employed patients, 20% (9/45) reported that their illness/treatment was not a hindrance to their job, 16% (7/45) stated that it was causing some symptoms, and 36% (16/45) pointed out that they (sometimes or often) had to slow down their working pace or change their work methods. Sixteen percent (7/45) stated that they could only do part-time work, and 27% (12/45) stated that they felt entirely unable to work. The median WAI in employed patients was 6/10 (range 0–10). Decisional conflict over the decision to receive chemotherapy was reported by 41% (17/42) of patients who were experiencing financial toxicity according to the EORTC QLQ-C30, whereas this was 30% (28/94) in patients who did not experience financial toxicity (*p* = 0.221). In one patient, the decisional conflict score was missing.

## Discussion

This analysis of a cohort of patients with advanced STS reported that even before starting first-line palliative chemotherapy, almost one-third of patients already experienced financial toxicity. The percentage of patients with clinically meaningful worse financial difficulty scores compared to normative data was twice as high in UK patients compared to NL patients. Additionally, predictors of financial toxicity varied between the two countries.

Financial toxicity in cancer patients has been evaluated previously in different settings [8, 36–38]. However, comparing financial toxicity across studies is challenging as different studies address different patient populations (e.g., long-term survivors, localized versus advanced disease, different cancer types), use different definitions of financial toxicity, and use different measurement tools. Furthermore, differences in general population norm data among countries and differences in health care settings (e.g., availability of a public health care system) restrict the comparison of financial toxicity among cancer patients across countries [34]. In one study evaluating QoL at progression in STS patients living in the US or Europe, financial difficulty measured with the EORTC QLQ-C30 scale showed a mean score of 25 (SD 33) at baseline and 30 (SD 34) at progression, which is consistent with our results (UK mean: 24; NL mean: 12) [39].

Looking at the general population norm data, the mean financial difficulty score is higher (i.e., worse) for UK residents (mean 15, SD 29) compared to NL residents (mean 5, SD 17). Our results show that these scores deteriorate when someone gets diagnosed with an advanced soft tissue sarcoma (UK: mean 24, SD 34; NL: mean 12, SD 25). The large standard deviation of the financial difficulty score in UK patients suggests that the UK population is very heterogeneous. In univariate analysis, UK residence was a negative predictor for financial toxicity. Multivariate analysis indicated that the higher scores observed in the UK are at least partially attributed to the income of the patient. This is not surprising, considering that more than 80% of patients were of working age (< 65 years). This aligns with a previous sensitivity analysis focusing on patients aged ≤ 60 years, showing that employment was the most important predictor of financial toxicity [24]. In the NL, neither income nor a change in employment status was identified as a predictor. This might be due to the organization of the Dutch healthcare system, where the first year of sick leave is paid at 100% of the salary by the employer and the second year at 70% of the salary, meaning that cancer patients in the Netherlands who were employed are financially well supported for 2 years after diagnosis. Additionally, the need for patients in the UK to travel greater distances to access palliative chemotherapy at sarcoma reference centers may play a role, as the UK has only nine reference centers for a population of 68.3 million, compared to seven centers in the Netherlands, which serves a population of 17.4 million [40, 41].

Univariate analysis showed that patients with a single relationship status and patients experiencing a change in employment status due to their cancer diagnosis were most at risk of financial toxicity. These findings align with previously published research on financial toxicity in cancer patients [8, 17, 36–38, 42]. Reduced working hours or inability to work may lead to the depletion of financial reserves required to meet direct and indirect medical costs. Single patients or patients living alone cannot rely on a partner for financial support, thereby increasing their susceptibility to financial toxicity.

In contrast, certain previously reported negative predictors, such as younger age, lower educational level, and extra costs (i.e., out-of-pocket costs), were not significant in our analysis. For example, although younger patients typically possess fewer financial reserves and often have a lower income, and several studies have demonstrated their vulnerability to financial toxicity [17, 38, 43], in our study, age was not identified as a predictor for financial toxicity, possibly due to sample size limitations and grouping of age categories.

In both the UK and NL, sarcoma care is centralized in sarcoma specialist centers. Consequently, it is not surprising that up to two-thirds of patients reported travel expenses. This suggests that patients with sarcoma or other rare tumors may need more financial support compared to patients with common cancers, or a care model that allows central coordination but local delivery of treatment. Strikingly, only a minority of patients indicated that they experience financial difficulties due to travel expenses. However, the impact of extra costs might be influenced by other factors such as household income and financial reserves [44]. Moreover, we did not collect data on the specific amount of extra costs, and extra costs were dichotomized into two categories, which might lead to an overestimation of the extent of extra costs.

Our results show that in addition to dealing with their sarcoma diagnosis, patients have to deal with financial toxicity, which can lead to feelings of being compelled to work, lifestyle adjustments, or uncertainty regarding treatment decisions (i.e., decisional conflict). For example, despite an interval between diagnosis of advanced STS and study participation of > 6 months in most patients, over half of working-age patients were still employed at the moment of study enrollment. One-third of working patients felt entirely unable to work, and the median workability index was only 6/10 (range 0–10). This observation might suggest that patients feel forced to work to avoid income loss. This hypothesis is supported by the univariate analysis, where a change in employment status emerged as a negative predictor for financial toxicity.

In both the UK and NL, patients participating in the study were insured by the public health insurance system. Therefore, our results highlight that public health insurance does not eliminate financial distress and its disparities among cancer patients. Understanding the patient’s experience of financial toxicity represents an unmet need. Patients might not always be aware of available financial support and, therefore, might not raise this issue themselves. More awareness of financial toxicity could be created by implementing screening for financial toxicity in clinical practice, as suggested by the expert consensus statement of the European Society of Medical Oncology (ESMO) [45]. For instance, the EORTC QLQ-C30 financial difficulty scale used in this study (“In the past week, has your physical condition or medical treatment caused you financial difficulties?”), is a practical tool for initial screening. Positive screening should be followed by an in-depth assessment of financial toxicity. This also holds implications for clinical practice and policy solutions. For example, if a patient suffers from financial difficulties, counseling by a professional (e.g., social worker) should be provided. Additionally, health insurance services could create awareness of possible financial benefits and provide information on how to claim them. Employers could offer work adjustments or support for patients who wish to continue or return to work. The government could support cancer patients by assuring financial support in cases of income loss. Additionally, there might be a stigma associated with claiming financial help, which could deter patients from seeking financial support. Addressing this stigma is crucial to ensure that patients feel comfortable accessing financial resources. Finally, it can help mitigate financial toxicity by creating more awareness of financial toxicity, for example, by asking if the cancer diagnosis or treatment leads to extra costs for the patient. This approach may help prevent treatment decisions from being influenced by financial burdens, especially when the treatment is expected to provide a clear benefit.

One of the major strengths of this study is that we included various sociodemographic characteristics and that results were compared with general country-specific population norm data, thereby reducing the amount of confounding. Another strength is that we used validated measurement tools (i.e. EORTC QLQ-C30) and thresholds for defining clinically meaningful changes [34]. A limitation is that due to the relatively small sample size, the statistical power to identify predictors of financial toxicity might have been limited. Furthermore, other factors potentially influencing financial toxicity, such as available savings and the absolute number of extra costs, were not collected. The cut-off of £/€2000 net salary/month is arbitrary, and we did not take into account differences in costs of living in the UK and NL. Furthermore, about 20% of patients preferred not to disclose their net income, possibly inducing bias. Another limitation is that the data were collected between 2018 and 2020, which may no longer fully reflect current circumstances. Factors that potentially impact financial toxicity, such as the organization of palliative care pathways and reimbursement policies, may have changed since the time of data collection.

## Conclusion

This is the first study assessing the prevalence and predictors of financial toxicity in patients with advanced STS starting palliative systemic therapy. Financial toxicity is common among patients diagnosed with advanced STS, even in countries with a public health care system, and can lead to additional stress during this challenging time. Identifying patients at risk of treatment-related financial toxicity may enable timely provision of support by different stakeholders, which may have a positive impact on overall quality of life.

## Data Availability

No datasets were generated or analysed during the current study.

## Appendix

**Table 4:** Sociodemographic characteristics

Sociodemographic characteristics			
	UK (*n* = 72)	NL (*n* = 65)	*P*-value
Age, years			0.966
18–39	6 (8)	5 (8)	
40–65	36 (50)	34 (52)	
> 65	30 (42)	26 (40)	
Disease timeline			**0.041**
≤ 6 months	25 (35)	34 (52)	
> 6 months	47 (65)	31 (48)	
Gender			0.609
Male	34 (47)	34 (52)	
Female	38 (53)	31 (48)	
Relationship status			0.820
Married/partner	59 (82)	55 (85)	
Single	13 (18)	10 (15)	
Education level			** < 0.001**
Low	18 (25)	9 (14)	
Medium	30 (42)	49 (75)	
High	24 (33)	7 (11)	
Disease extent			0.374
Locally advanced	8 (11)	4 (6)	
Metastatic	64 (89)	61 (94)	
Histological subtype			0.236
Leiomyosarcoma	25 (35)	15 (23)	
Liposarcoma	15 (21)	15 (23)	
UPS	11 (15)	6 (9)	
Angiosarcoma	3 (4)	7 (11)	
Other	18 (25)	22 (34)	
